# Evolutionary psychiatry: a new College special interest group

**DOI:** 10.1192/pb.bp.115.052407

**Published:** 2016-10

**Authors:** Riadh Abed, Paul St John-Smith

**Affiliations:** 1 Mental Health Tribunal Service, Sheffield, UK; 2 Hertfordshire Partnership University NHS Foundation Trust

## Abstract

Evolutionary science remains an overlooked area in psychiatry and medicine. The newly established Royal College of Psychiatrists' Evolutionary Psychiatry Special Interest Group aims to reverse this trend by raising the profile of evolutionary thinking among College members and others further afield. Here we provide a brief outline of the importance of the evolutionary approach to both the theory and practice of psychiatry and for future research.

The significance of evolutionary theory to the biomedical sciences generally remains underappreciated among psychiatrists and medics.^1^ Evolutionary science does not currently feature in the undergraduate curriculum of most medical schools in the UK, nor is it part of the syllabus of the Royal College of Psychiatrists' membership examinations (MRCPsych). As a result, most psychiatrists remain largely unaware of the relevance of evolution to mental disorder and dysfunction.

Unlike evolutionary psychology, which is a vibrant and thriving sub-discipline of academic psychology with a strong and well-funded research programme, evolutionary psychiatry remains the interest of a small number of psychiatrists who are thinly scattered across the world. Despite the publication of a number of excellent evolutionary psychiatry and evolutionary medicine texts over the past two decades,^2–7^ as well as numerous scholarly peer-reviewed articles, there is still neither a university academic department nor an academic journal anywhere in the world dedicated to researching and promoting the evolutionary approach to psychiatry.

We are therefore greatly encouraged by the Royal College of Psychiatrists' Council's decision to support the formation of a new evolutionary psychiatry special interest group and even more encouraged that a significant number of members and fellows of the College voted in favour of this initiative. This comes 2 years after the World Psychiatric Association (WPA) approved the setting up of the WPA Section on Evolutionary Psychiatry.

## What the evolutionary approach may offer

Why is the evolutionary approach relevant to psychiatry? To date, psychiatry has operated without an accepted unifying framework and has been characterised by a plurality of approaches, some of which are diametrically at odds with each other.^3^ This pluralism may be presented by some as a sign of strength and vibrancy but it is more likely in our view to be a sign of conceptual weakness. This weakness is exemplified by the lack of the most rudimentary rules about the function of the human mind. The consequences of this state of unconstrained pluralism are that any theory, however irrational, can demand equal attention.^8^

Evolutionary science helps resolve these issues as it recognises two categories of causation: proximate (mechanism and ontogeny) and ultimate or evolutionary (phylogenetic and function).^9^ These are reflected in Tinbergen's four questions (Box 1). Proximate causes are the answer to the ‘how’ question and are the primary focus of non-evolutionary science, whereas ultimate causation is the answer to the ‘why’ question. Ultimate or evolutionary causation is a perspective which is unique to evolutionary science and theories of ultimate causation will be compatible with a whole range of proximate causes. Neuroscience has made significant advances in exploring the proximate causes of psychopathology but relatively few studies have addressed evolutionary or ultimate causes of traits or disorders.^10^ For example, the finding that familial adversity during early life leads to early sexual maturity in females can be explained through studying the hormonal, biochemical and other physiological mechanisms that bring about early puberty (proximate causes). However, the evolutionary or ultimate causation of this phenomenon that has been replicated across a number of species and appears to be a widespread mammalian capacity^11^ suggests that this acceleration is likely to be an adaptive response to the organism's ‘prediction’ of future adversity from current or childhood adversity. Thus, those females who possessed the capacity for phenotypic plasticity (the ability to vary their phenotype by maturing early) were more reproductively successful than those who lacked this capacity.^11,12^ This example illustrates the fallacy of considering nature or nurture in isolation and shows that nurture clearly works via nature,^13^ in this case – adversity working through particular genes that allow for phenotypic plasticity. It also highlights the importance of ‘life history strategy’ (in this case, by switching to a fast life history strategy) in shaping human behavioural patterns and in creating vulnerabilities to certain mental disorders.^14^ More importantly, however, this example demonstrates that considering biological phenomena from an evolutionary perspective can give rise to insights and generate hypotheses (testable and refutable) that would not otherwise be possible. In other words, it shows that considering the ultimate causation of a biological phenomenon can uncover areas of biological enquiry that would have otherwise remained invisible.

**Box 1** Tinbergen's four questions

1. What are the mechanisms that cause the biological phenomenon?
2. How does that develop in an individual (ontogenesis)?
3. How did it evolve (phylogenetic history)?
4. What was the function and fitness value of the trait/system/behaviour?

Another illustration of the effectiveness of the evolutionary approach was in the area of child abuse, by uncovering what Daly & Wilson have termed the ‘Cinderella effect’.^15,16^ As predicted by Hamilton's theory of kin selection,^17^ they demonstrated that children were at significantly greater risk of abuse when living with a step-parent compared with two genetic parents. They also found that pre-school stepchildren were 40–100 times at greater risk of homicide than when they lived with two genetic parents. They concluded that step-parentage was the single most important risk factor for child abuse in pre-school children. It is notable that non-evolutionary researchers had not thought to examine these questions and is an example of a hypothesis of ultimate causation (that costly parental investment is unlikely to be indiscriminate) guiding research.

Understanding how evolution operates helps clinicians realise that evolution (selection) does not design disease or disorder but rather creates the vulnerability to disorder.^5^ These vulnerabilities arise from the very nature of the evolutionary process where selection (natural, sexual, social, etc.) operates to benefit reproduction, not health, where design involves significant trade-offs, and where biological evolution always lags behind cultural evolution leading to ‘mismatch’.^18^ Also, neglecting the evolutionary perspective often leads to clinicians confusing an emotional defence with a disorder.^19^ For example, the aversive emotions of anxiety and depression are designed by selection to act as defences that protect us from risks that, when appropriately activated, can improve our chances of survival and reproduction. Furthermore, these defences, like the experience of pain, are designed to cause distress and discomfort to ensure that they effectively prevent the risk of greater harm. Therefore, equating distress with disorder is to fundamentally misunderstand the function of our emotional defences.^20^

## Examples of the use of the evolutionary approach

Nesse^21^ has proposed an influential evolutionary model to explain the way that defences, designed by selection, can become (or be perceived as) disorders. He calls this the ‘smoke detector principle’. Many of the body's adaptive responses, such as cough, pain, anxiety and depression, are crucial defences that remain latent until they are aroused by cues that indicate the presence of a threat. Natural selection has shaped the species' mechanisms to express these defences optimally in situations where their benefits exceed their costs. However, they are designed in such a way as to allow for many false alarms, as the cost of a failure to activate can be catastrophic. The consequent oversensitivity, coupled with the novel modern human environments where the level of risk is much reduced compared with the ancestral environment, leads to many false positive responses and thus much unnecessary suffering. This process is analogous to the design of smoke detectors where the cost of a false positive is trivial compared with a failure to detect a real fire. Given that there are many more false alarms than real ones, it is usually clinically safe to block the response. However, this is not invariably the case and there will be situations where the individual (patient) is facing real-life threats to their well-being. Hence, this perspective can guide clinical decision-making as to when it would be safe to switch off the defensive response. It can also guide research into anxiety and depression, both in the general population and in clinical settings where the proportion of those whose defences are appropriately activated (as opposed to false alarms) is currently unknown.

We suggest that evolutionary psychiatry offers a number of definite advantages to psychiatry.^22^ These include:

- asking new questions about why evolution has left us all vulnerable to mental disorders^23^ (Box 2)
- providing a way to think clearly about development and the ways that early experiences influence later characteristics
- providing a foundation for understanding emotions and their regulation
- providing a foundation for a scientific diagnostic system
- providing a framework for incorporating multiple causal factors that explain why some people get mental disorders while others do not.^22^

**Box 2** Pathways that mediate the influence of evolutionary processes on disease vulnerability

- Mismatch: exposure to evolutionarily mismatched or novel environment
- Life history factors
- Excessive defence mechanisms
- Co-evolutionary considerations: losing the arms race against pathogens
- Constraints imposed by evolutionary history
- Sexual selection and its consequences
- Balancing selection: maintaining an allele that raises disease risk
- Demographic history and its consequences
- Selection favours reproductive success at the expense of health

Adapted from Gluckman *et al.*^6^

There are numerous examples for the application of the evolutionary approach to mental disorder. These include the cortical dysconnectivity theory of the social brain for schizophrenia,^24^ the social competition theory for depression,^25^ the pleiotropic theory of human senescence,^26^ and the sexual competition theory for eating disorders,^27^ to mention just a few. In addition, evolutionary theory can have direct benefits to patients. A prime example is compassion-focused therapy,^28,29^ an evidence-based psychological therapy that has its roots in Bowlby's attachment theory. Compassion-focused therapy is based on a model of human emotions that recognises that emotions have evolved to fulfil a function and that human emotional systems have a phylogenetic (evolutionary) history as well as developmental and contextual dimensions. The emotion of compassion that has its origins in the human (and the more ancient, mammalian) capacity to care for our young has been applied over human history to other members of the kin group and subsequently to other in-group members. The healing qualities of compassion that have such profound effects on the emotional and personality development of humans during early life are employed by the therapist in compassion-focused therapy, with the aim of promoting recovery through helping patients develop the capacity for self-compassion.

## Evolutionary science can help clarify the concept of mental disorder

We would propose that evolution can be usefully viewed as a meta-theory of psychiatry that provides a framework that helps define the role of various proximate factors in the causation of mental disorder. Hence, accepting the assumption that the human mind/brain has been shaped by selection over thousands of generations to solve the recurring problems of survival and reproduction can in itself provide a plausibility index that renders a whole range of hypotheses unlikely while pointing towards the possibility of others. We would suggest, therefore, that evolutionary science has the capacity to provide psychiatry with a much needed plausibility or reality check that can favour promising hypotheses while providing an early warning regarding those that are likely to lead to scientific blind alleys.

It was Lorenz who, in 1937, demonstrated that behavioural patterns can be designed by evolutionary processes in exactly the same way as anatomical structures,^30^ and yet many psychiatrists continue to equate the biological exclusively with genetic and neurochemical processes. The evolutionary approach can help to show that the molecular level of brain functioning is no more or less biological than the macrobiological level or the level of organismic behaviour.^31^ It is worth remembering that the end products of the human mind/brain are behaviour patterns, emotions and cognitions and therefore these are the phenotypic characteristics of our brains that have been shaped by selection, whereas the brain circuitry and neurotransmitters are subservient systems that have evolved to generate those end products. It follows therefore that human behavioural strategies are fundamentally biological phenomena that can only be fully understood within their correct social and environmental context. Hence, it becomes legitimate to speak of a biology of human social behaviour, a biology of culture and so forth.

Wakefield^32,33^ proposed that a Darwinian concept of mental disorder builds on two basic ideas. The first is impairment in the capacity of the individual to achieve important biological goals and the second is that an individual's functional capacity cannot be assessed without consideration of the environment in which they live. Furthermore, he suggested that mental disorder is a state of harmful dysfunction that is caused by a failure of a biological mechanism to perform its evolved function and that it causes harm or damage as judged by sociocultural standards. Although Wakefield's concept of mental disorder raises problems of its own, it represents a significant advance over the current atheoretical definitions.

## The Evolutionary Psychiatry Special Interest Group and what it might offer

We are hoping that the support that the establishment of the special interest group has received from the College membership will translate into support for its activities over the coming months and years. We are hoping that this will encourage evolutionary inspired research, help produce and distribute teaching material on evolutionary principles, help advocate for the inclusion of evolution into the undergraduate medical curriculum in the UK and elsewhere as well as into the MRCPsych syllabus, in addition to organising workshops, symposia and conferences.

We are aware that evolutionary psychiatry has its vocal critics and detractors. We do not believe that it is possible to convince those who object to evolution on ideological or religious grounds. However, we fully understand and even sympathise with the position of those whose objections arise from concerns regarding the need to maintain high standards of scientific rigour and the avoidance of ‘just-so stories’. We would suggest, however, that some of the most prevalent just-so stories have nothing to do with evolution and include assertions such as that all mental disorders are diseases or alternatively that mental illness is a myth. We believe that evolutionary inspired theories and hypotheses must ultimately be supported, refuted (and discarded) or modified on the basis of empirical evidence and not through dogma or appeal to authority.

We suggest that without a broad, interactionist, evolutionarily grounded approach, psychiatric trainees are likely to feel bewildered by and discouraged from exploring other modes of scientific investigation and understanding, to the detriment of their patients and their own professional satisfaction. However, although using the evolutionary model encourages eclecticism and considers how brain/mind is influenced by and influences a whole range of biopsychosocial-cultural issues, we should not seek eclecticism at any evidential cost.

## Conclusions

Among Darwin's lasting legacies is our knowledge that the human brain/mind evolved through evolutionary processes. The human brain consumes around 20% of the body's energy intake while constituting merely 2% of its weight. Such an organ would not have evolved if it had not performed some vital adaptive functions in our evolutionary past. The challenge for evolutionary psychiatry is to move from general facts to evidentially well-supported specifics about the adaptive processes that shaped the mind and thus created the vulnerability to illness. It may be that there are many things about the evolution of the human mind that we will never know and about which we can only hypothesise. At its very best, however, it can aid the discovery of knowledge of why all our complex human psychological characteristics evolved, why we have vulnerabilities to illness and ultimately, what we might do about that in terms of prevention and treatment.
